# Characterization of Screen-Printed Organic Electrochemical Transistors to Detect Cations of Different Sizes

**DOI:** 10.3390/s16101599

**Published:** 2016-09-28

**Authors:** Laura Contat-Rodrigo, Clara Pérez-Fuster, José Vicente Lidón-Roger, Annalisa Bonfiglio, Eduardo García-Breijo

**Affiliations:** 1 Group of Electronic Development and Printed Sensors (GEDPS), Centro de Reconocimiento Molecular y Desarrollo Tecnológico, Unidad Mixta UPV-UV, Valencia 46022, Spain; lcontat@ter.upv.es (L.C.-R.); cperezf@eln.upv.es (C.P.-F.); jvlidon@eln.upv.es (J.V.L.-R.); 2 Dipartimento di Ingegneria Elettrica ed Elettronica, Università di Cagliari, Piazza d’ Armi, Cagliari 09123, Italy; annalisa@diee.unica.it

**Keywords:** organic electrochemical transistors, screen-printing, PEDOT:PSS, large-size cations

## Abstract

A novel screen-printing fabrication method was used to prepare organic electrochemical transistors (OECTs) based on poly(3,4-ethylenedioxythiophene) doped with polysterene sulfonate (PEDOT:PSS). Initially, three types of these screen-printed OECTs with a different channel and gate areas ratio were compared in terms of output characteristics, transfer characteristics, and current modulation in a phosphate buffered saline (PBS) solution. Results confirm that transistors with a gate electrode larger than the channel exhibit higher modulation. OECTs with this geometry were therefore chosen to investigate their ion-sensitive properties in aqueous solutions of cations of different sizes (sodium and rhodamine B). The effect of the gate electrode was additionally studied by comparing these all-PEDOT:PSS transistors with OECTs with the same geometry but with a non-polarizable metal gate (Ag). The operation of the all-PEDOT:PSS OECTs yields a response that is not dependent on a Na^+^ or rhodamine concentration. The weak modulation of these transistors can be explained assuming that PEDOT:PSS behaves like a supercapacitor. In contrast, the operation of Ag-Gate OECTs yields a response that is dependent on ion concentration due to the redox reaction taking place at the gate electrode with Cl^−^ counter-ions. This indicates that, for cation detection, the response is maximized in OECTs with non-polarizable gate electrodes.

## 1. Introduction

Organic electronics has been extensively developed since the discovery of conducting polymers in the late 1970s [1,2] due to the unique features that these materials can offer, such as low-cost fabrication, low temperature processing, mechanical flexibility, ionic conductivity, and facile chemical modification [3]. As a result, many electronic devices based on organic semiconductors have been developed, including organic light emitting diodes (OLEDs) [4], organic photovoltaics (OPVs) [5], and organic thin film transistors (OTFTs) [6,7]. Among the latter, organic electrochemical transistors (OECTs) have attracted considerable interest in recent years for their application as organic semiconductor devices in many fields, especially for chemical and biological sensing due to their ability to operate in aqueous environments [8,9].

OECTs were initially reported by White in the 1980s [10]. The essential components of an OECT are an organic semiconductor film (in its doped-conducting-state), the channel, with source and drain electrodes, and an electrolyte bridging the channel and the gate electrode. As a convention, the source electrode is grounded and a voltage is applied to the drain electrode relative to the ground. The operation of OECTs is based on the modulation of the channel current by electrochemical doping or de-doping from the electrolyte when gate voltages are applied.

One of the most widely used conducting polymers in these types of devices is poly(3,4-ethylenedioxythiophene) doped with polysterene sulfonate (PEDOT:PSS) [11,12]. PEDOT:PSS is a degenerately doped p-type organic semiconductor that is commercially available and can be readily prepared by using conventional solution processing techniques [13,14,15]. In addition, it exhibits high conductivity, excellent thermal stability, and good stability in a wide pH range. Upon the application of a positive gate voltage in PEDOT:PSS-based OECTs, cations from the electrolyte medium migrate into the conducting polymer [16,17]. This in turn de-dopes PEDOT:PSS and leads to a decrease in the channel current. De-doping occurs by reduction of the highly conducting form of PEDOT^+^ to the less conducting form of PEDOT^0^ [18], according to the following electrochemical reaction:

(1) $$n\left(PEDOT^{+}:PSS^{-}\right)+M^{n+}+ne^{-}⇆nPEDOT^{0}+M^{n+}:nPSS^{-},$$

where *M^n+^* is a cation in the electrolyte medium, *n* is the number of charge of the cation, and *e^−^* is an electron from the source electrode. When the gate voltage is removed, cations migrate back into the electrolyte medium, and the original conductivity of the organic semiconductor is restored.

As a result of this working principle, the characteristics of OECTs based on PEDOT:PSS are usually sensitive to the ion concentration in the electrolyte. On this basis, these devices have been successfully employed as chemical and biological sensors [17,19]. A comprehensive description of the ion-sensitive behavior of these devices is therefore critical for their optimization for sensing applications. Lin first studied the ion-sensitive properties of these types of devices in aqueous solutions with different cationic species (H^+^, K^+^, Na^+^, Ca^2+^, and Al^3+^), finding that the transfer curves shift to lower gate voltage horizontally with the increase in the concentration of cations [19]. This shift was also found to be dependent on the type of ion in the electrolyte, decreasing with the increase in the ionic charge.

More recently, Malliaras et al. reported on the mobility of various cations in PEDOT:PSS films, proving that this polymer is an efficient ion transporter, not only of small cations such as H^+^, K^+^, and Na^+^, but also of choline (C_5_H_14_NO^+^), a biologically-relevant ion of larger size [20]. However, efficient transport of large ions through this material has not been yet reported, despite the interest of knowing the maximum detectable size of chemical and biological analytes for sensing applications.

In OECTs based on PEDOT:PSS, the gate electrode also plays an important role with respect to its ion-sensitive properties. Lin demonstrated that, for devices with Ag/AgCl gate electrodes, Nernstian relationships are found, whereas for devices with metal gate electrodes (Pt and Au), the ion sensitivity is higher than that given by the Nernst equation [19].

The device geometry is another critical issue to the ion-sensitive behavior of OECTs based on PEDOT:PSS. Optimization of the performance of these devices can be achieved by changing the ratio between the channel and gate areas, and the channel geometry (width and length) [21,22].

The practical implementation of these different geometries ultimately depends on the processing techniques that are used for the fabrication of these transistors. Many innovative techniques have been developed in the last decade for the fabrication of OECTs. In particular, there has been a significant increase in the interest in using printing techniques, since these provide a wide range of advantages compared to traditional fabrication techniques of silicon-based electronics. One of the most important advantages is the possibility of working with flexible substrates. Furthermore, printing techniques (inkjet, gravure, serigraphy, etc.) can be combined with high-throughput roll-to-roll manufacturing techniques (screen-printing, flexography, etc.) to produce low-cost organic electronic devices. Although printing techniques offer many advantages, only a few examples of the fabrication of OECTs using these technologies are reported in the literature [15,23,24]. In general, these types of transistors are prepared using alternative and more expensive techniques, such as lithography [16,21,22,25,26].

In this communication, we report on the characterization of various PEDOT:PSS-based OECTs prepared using a screen-printing technique over a flexible substrate. Screen-printed transistors with different geometries are compared in terms of their electrical characteristics and current modulation. In addition, the effect of the gate electrode on the ion-sensitive properties of these OECTs in aqueous solutions of cations of different sizes is also investigated.

## 2. Materials and Methods

**Preparation of the phosphate buffered saline (PBS) solution:** A 0.1-M PBS solution was prepared by dissolving K_2_HPO_4_ (Scharlau, Barcelona, Spain) and KH_2_PO_4_ (Probus, Badalona, Spain) in Milli-Q water. The PBS solution had a pH of 6.8, as measured by a pH meter.

**Preparation of NaCl solutions:** Aqueous solutions containing sodium cations (Na^+^) were prepared by dissolving sodium chloride (Scharlau) in Milli-Q water with different molar concentrations (10^−3^ M, 10^−4^ M, and 10^−5^ M).

**Preparation of rhodamine solutions:** Aqueous solutions containing rhodamine B cations (C_28_H_31_N_2_O_3_^+^) were prepared by dissolving rhodamine B (C_28_H_31_ClN_2_O_3_) (Sigma-Aldrich, Madrid, Spain) in Milli-Q water with different molar concentrations (10^−3^ M, 10^−4^ M, and 10^−5^ M).

**Transistor fabrication:** For the fabrication of the OECTs, PEDOT:PSS was screen-printed over polyester film. Screen-printing was performed with a polyester mesh screen PET 1500 165/420-34W/32 µm (SEFAR, Barcelona, Spain) and an UV film DIRASOL 132 (FUJIFILM, Barcelona, Spain). The final screen thickness was 55 µm. The OECTs pattern was transferred to screen by using an UV light. PEDOT:PSS CLEVIOS S V3 (Heraeus, Madrid, Spain) (Table 1) was mixed with ethylene glycol (4:1 by volume). The mixture was re-dispersed for 1 h at 1000 rpm before printing. Printing was carried out by using AUREL 900 with a 75º shore squeegee hardness, 1 bar force, and 0.2 m/s. The substrate used was a transparent and flexible polyester MELINEX ST506 (thickness of 175 µm, DuPont Teijin Films, Scotland, UK) cleaned with acetone, ethanol, and deionized water. Finally, the OECTs were cured in an air oven at 80 °C for 5 min.

**Electrical measurements:** All measurements were performed using a Keithley 4200 semiconductor characterization system.

**Cyclic voltammetry experiments:** The electrochemical characterization was carried out with an Autolab PGSTAT12 potentiostat/galvanostat electrochemical analysis system.

## 3. Results and Discussion

### 3.1. Electrical Characterization: Effect of the Device Geometry

**Figure 1:**
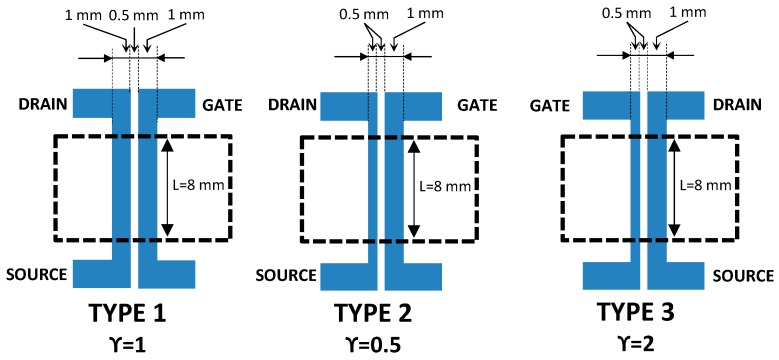
Schematic representation of the devices layout with three different ratios between the channel and gate areas.

Three types of all-PEDOT:PSS screen-printed transistors with different geometries were prepared by modifying the ratio between the channel and gate areas A_ch_/A_g_ (γ) (where A_ch_ is the channel area and A_g_ is the gate area) (Figure 1). Type 1 OECT is a symmetric device with γ = 1. In contrast, type 2 and type 3 OECTs are asymmetric devices with γ = 0.5 and γ = 2, respectively.

These three types of all-PEDOT:PSS OECTs were initially characterized in a PBS solution. Electrical measurements were performed, filling the electrolyte reservoir with 150 μL of aqueous 0.1-M PBS. The output characteristics, the transfer characteristics, and the current modulation of each type of OECT were obtained and compared.

**Figure 2:**
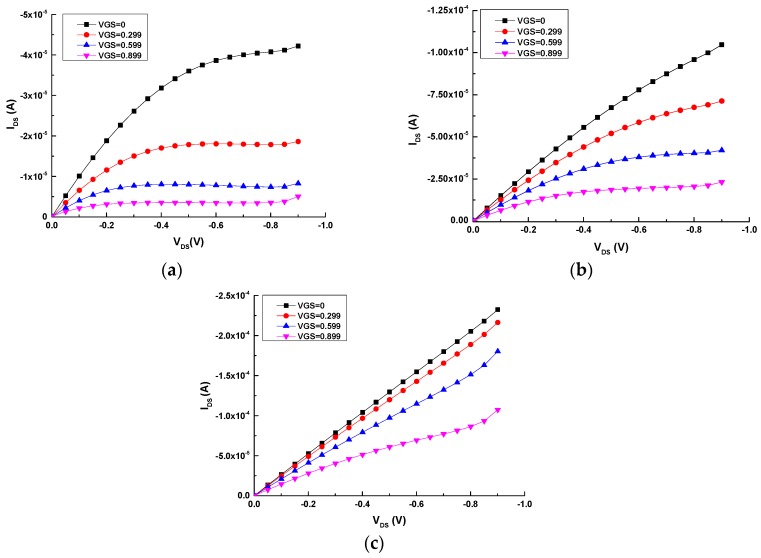
Output characteristics of the three types of all-PEDOT:PSS OECTs: (**a**) Type 2 (γ = 0.5); (**b**) Type 1 (γ = 1); (**c**) Type 3 (γ = 2).

Figure 2 shows the output curves of the all-PEDOT:PSS transistors with different channel and gate areas. It can be observed that the geometry has a large influence on the output characteristics of these OECTs. Results clearly show that the transistor with a small area ratio (γ = 0.5) exhibits a strong modulation of the drain current (I_DS_) (Figure 2a), while the modulation of this current is weaker for the transistor with a large area ratio (γ = 2) (Figure 2c) [22].

**Figure 3:**
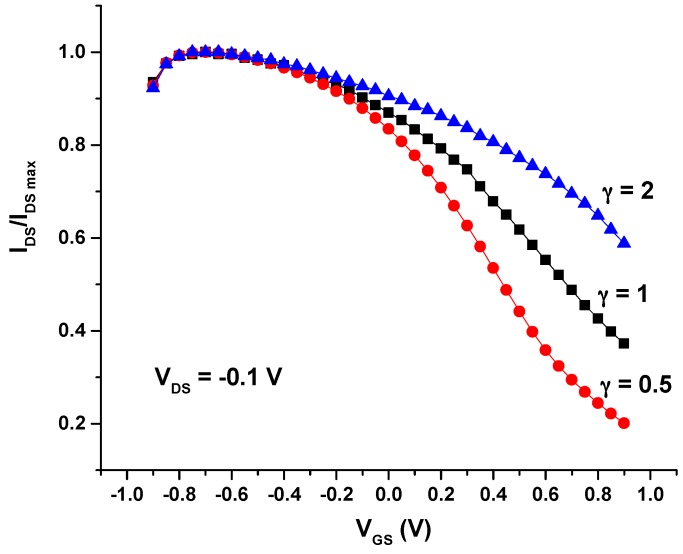
Normalized I_DS_ vs. V_GS_ curve for the three types of all-PEDOT:PSS OECTs.

Concerning the transfer characteristics of these screen-printed OECTs, these were obtained plotting the drain current (I_DS_) (normalized to its maximum value) vs. the gate voltage (V_GS_) from −0.9 V to 0.9 V under a constant drain voltage (V_DS_) (Figure 3). It can be noticed that the maximum (on) current is reached for negative values of V_GS_, indicating that negative gate-source voltage leads to a further doping of PEDOT:PSS in the channel [15,23]. Moreover, it is observed that the offset voltage (V_off_) depends on the geometry, as for metal-gated structures.

**Figure 4:**
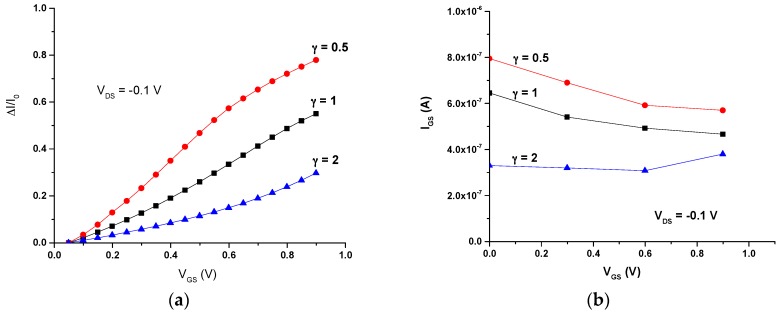
(**a**) Current modulation ΔI/I_0_ vs. V_GS_; (**b**) I_GS_ vs. V_GS_.

Finally, the current modulation of these all-PEDOT:PSS OECTs was determined. Plots of ΔI/I_0_ (ΔI/I_0_ = (|I − I_0_|/I_0_)), where I is the off current (V_GS_ ≠ 0) and I_0_ is the on current (V_GS_ = 0), vs. the gate voltage (V_GS_) for different values of γ are shown in Figure 4a. The different responses for the OECTs with different geometries are highlighted. The OECT with the smallest γ (large gate) exhibits the highest modulation throughout all the investigated V_GS_ range, while the OECT with a large γ (large channel) exhibits little modulation (Figure 4a).

This can be explained by the idea suggested by Malliaras [16,22] that, for these types of transistors, the electrolyte potential (*V_sol_*) is related to the area geometry (γ) according to

(2) $$V_{sol}=\frac{V_{GS}}{1+\gamma }.$$

**Figure 5:**
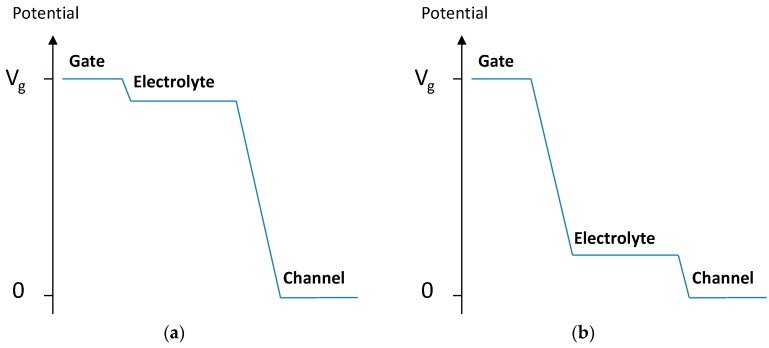
Potential distribution between the gate electrode and the channel for (**a**) γ = 0.5; (**b**) γ = 2.

In OECTs with a small γ, the electrolyte is nearly at the same potential as the gate, resulting in strong modulation. In contrast, in OECTs with large γ, the modulation is weak due to the large potential drop at the gate-electrolyte interface (Figure 5).

Equation (2) represents a non-Faradaic response. Demelas and co-workers demonstrated that an all-PEDOT:PSS OECT behaves similarly to a device with a gate metal working as a polarizable electrode, at least within a certain range of the V_GS_ bias. Therefore, these devices can be controlled by varying the geometry (i.e., the area ratio, γ) [23]. In particular, it is suggested that OECTs with a gate electrode that is much larger than the channel (a small γ) are used [22], which is in good agreement with the results presented here.

On the other hand, results also show that the gate-source current (I_GS_) is higher for the transistor with a small ratio between the channel and gate areas (Figure 4b).

### 3.2. Ion-Sensitivity: Effect of the Gate Electrode

According to the above results, the all-PEDOT:PSS OECT with a small γ (type 2, γ = 0.5) and better current modulation was chosen to investigate hereinafter the ion-sensitive properties of these transistors. For operation as an ion-to-electron converter, besides the use of OECTs with a small γ, it is also suggested that a non-polarizable gate (i.e., Ag) is alternatively used [22]. Therefore, the effect of the gate electrode on the performance of these transistors was additionally studied by comparing the response in aqueous solutions of cations of different sizes of the all-PEDOT:PSS transistor type 2 with an OECT with the same channel-to-gate area ratio (γ), but with a Ag gate.

**Figure 6:**
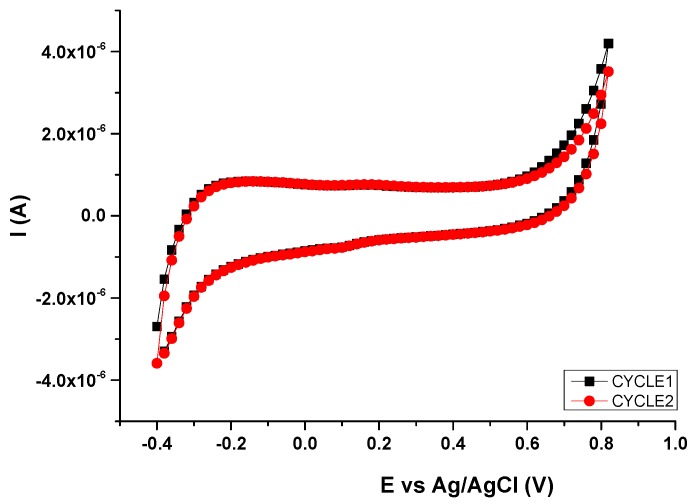
Electrochemical characterization of PEDOT:PSS. CV curves recorded at 0.02 V·s^−1^.

First, the gate voltage (V_GS_) range of these ion-sensitivity measurements was defined by performing a voltammetry of the PEDOT:PSS ink used for the fabrication of these OECTs (CLEVIOS S V3, Heraeus). A PEDOT:PSS electrode with the same dimensions and shape of the channel area served as the working electrode (WE) in a three-electrodes cell. A platinum wire was used as the counter electrode (CE), while all potentials were reported with the reference to the Ag/AgCl electrode (RE). Tests were performed in a 0.1-M PBS solution. Results evidence the redox behavior of this organic semiconductor (Figure 6). PEDOT:PSS undergoes reduction at approximately −0.4 V and oxidation near +0.8 V. According to these results, the study of the ion-sensitive properties of these OECTs was performed using a V_GS_ range from 0 V to 0.5 V.

**Figure 7:**
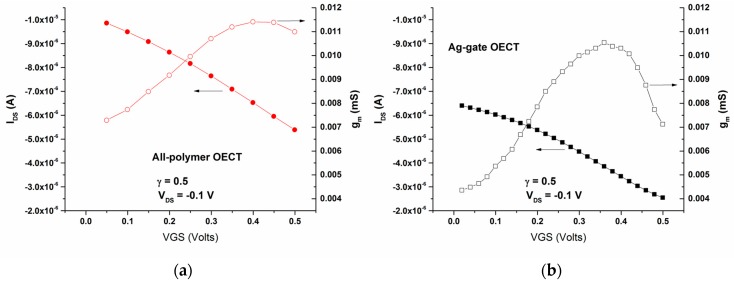
Transfer curves for V_DS_ = −0.1 V and their associated transconductances of (**a**) the all PEDOT:PSS OECT (type 2) and (**b**) the Ag-Gate OECT.

In addition, in this V_GS_ range, the transfer curves and the associated transconductances of the all-PEDOT:PSS OECT type 2 and the Ag-Gate OECT with the same γ were obtained in the PBS solution (Figure 7). The drain current (I_DS_) is shown in the transfer curves to decrease with the gate voltage for both OECTs, which is consistent with the operation in the depletion regime and is in good agreement with the operation mechanism of PEDOT:PSS-based OECTs [16,17]: upon the application of a positive gate voltage, cations from the electrolyte migrate into the conducting polymer. This de-dopes PEDOT:PSS and leads to a decrease in the drain current as shown in Figure 7. On the other hand, the transconductance is shown to reach a maximum value of g_m_ = 0.0115 mS at V_GS_ = 0.45 V for the all-PEDOT:PSS OECT, and of g_m_ = 0.0105 mS at V_GS_ = 0.35 V for the Ag-Gate OECT (Figure 7).

#### 3.2.1. Ion-Sensitivity towards Sodium Cations

**Figure 8:**
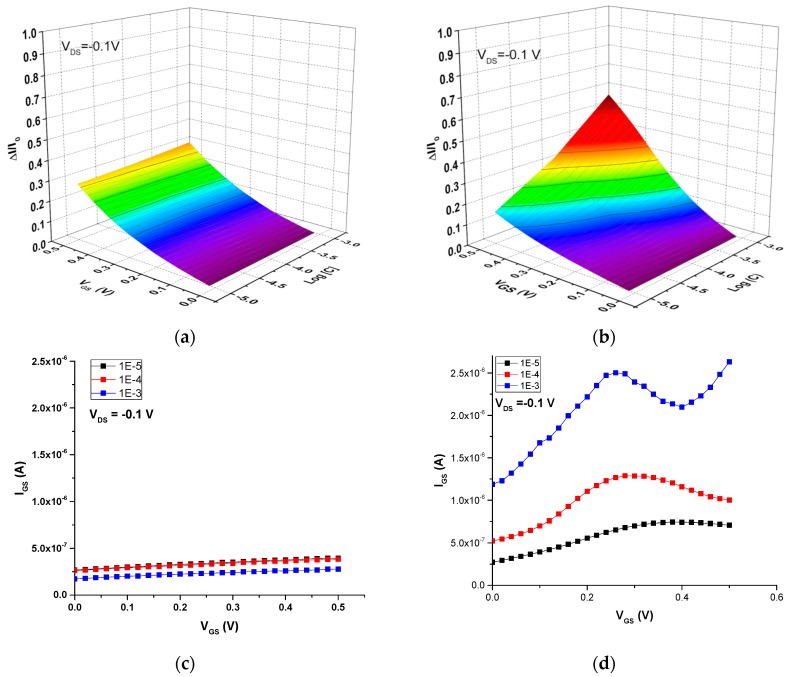
OECT response in NaCl aqueous solutions with different concentrations. Current modulation ΔI/I_0_ vs. V_GS_ for (**a**) all-PEDOT:PSS OECT; (**b**) Ag-Gate OECT. Plots of I_GS_ vs. V_GS_ for (**c**) all-PEDOT:PSS OECT; (**d**) Ag-Gate OECT.

Initially, the ion-sensitive properties of these screen-printed OECTs were studied in aqueous solutions of NaCl to investigate the detection of Na^+^, a biologically relevant small cation. Figure 8 shows the current modulation (ΔI/I_0_) and gate-source current (I_GS_) vs. the gate voltage (V_GS_) as a function of NaCl concentration for both the all-PEDOT:PSS OECT and the Ag-Gate OECT. The operation of the all-PEDOT:PSS OECT leads to a response that is not dependent on NaCl concentration. The drain current does not depend on concentration, which suggests that the electrolyte potential has diminished. As a result, these transistors exhibit a small potential drop between the electrolyte and the channel, which in turn translates to a weak current modulation (Figure 8a). Moreover, the drain current is close to its maximum value, which can indicate that only few Na^+^ cations have migrated into the channel. On the other hand, Demelas and coworkers have suggested that an all-PEDOT:PSS behaves similarly to a device with a polarizable gate (i.e., Pt), working in a non-Faradaic regime (as proven here by the very low gate current) [23] (Figure 8c). These authors also suggest that, in these types of transistors, PEDOT:PSS behaves like a supercapacitor, which could explain the weak dependence on the ionic concentration that is observed.

In contrast, the operation of the Ag-Gate OECT yields a response that is dependent on NaCl concentration. This indicates that, in this case, there is a large potential drop between the electrolyte and the channel, which in turn leads to a strong modulation of the drain current (Figure 8b). This can be explained by the redox reaction taking place at the Ag gate due to the presence of Cl^−^ counter-ions. As a consequence, there is almost no potential drop at the gate-electrolyte interface. The transistor operates in a Faradaic regime, as shown by the large gate current, which increases as the ionic concentration increases from 10^−5^ M to 10^−3^ M, due to the oxidation of the Ag gate (Figure 8d):

(3) $$Ag+Cl^{-}⇆AgCl+e^{-}.$$

#### 3.2.2. Ion-Sensitivity towards Rhodamine B Cations

**Figure 9:**
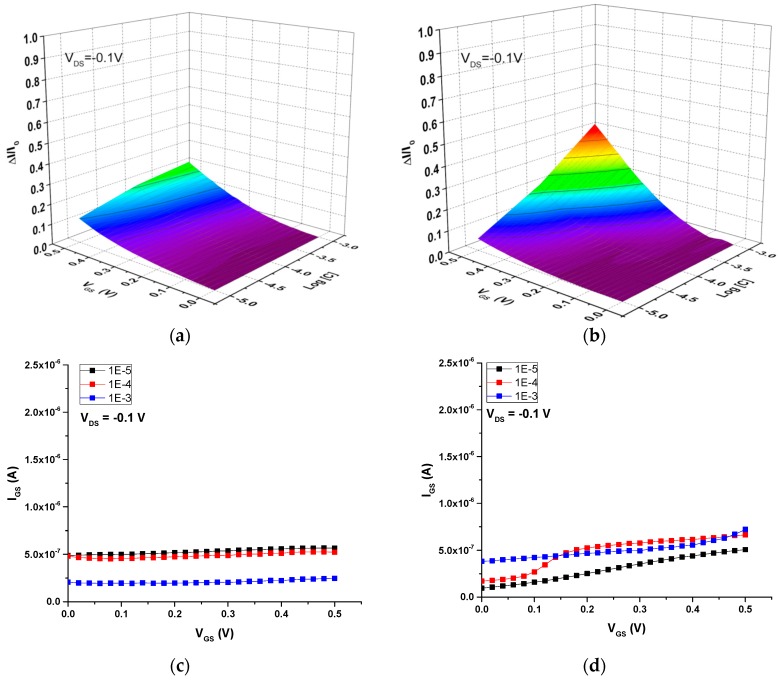
OECT response in rhodamine B aqueous solutions with different concentrations. Current modulation ΔI/I_0_ vs. V_GS_ for (**a**) all-PEDOT:PSS OECT; (**b**) Ag-Gate OECT. Plots of I_GS_ vs. V_GS_ for (**c**) all-PEDOT:PSS OECT; (**d**) Ag-Gate OECT.

Next, the ion-sensitive properties of these two OECTs were investigated in aqueous solutions of rhodamine B (C_28_H_31_N_2_O_3_^+^), a large-size cation widely used as a dye in biological applications due to is fluorescent properties. Figure 9 shows the current modulation (ΔI/I_0_) and I_GS_ vs. V_GS_ curves as a function of rhodamine concentration for both the all-PEDOT:PSS OECT and the Ag-Gate OECT. The operation of these two OECTs in rhodamine solutions yields to a response that is similar to that obtained in NaCl solutions. The drain current of the all-PEDOT:PSS OECT does not depend on concentration and is similar to its maximum value, which indicates that few rhodamine cations have migrated into the channel (Figure 9a). This weak modulation of the current could again be explained assuming that PEDOT:PSS behaves like a supercapacitor [23]. Compared to the operation of this transistor in NaCl solutions, the response is even lower in this case, suggesting that the migration of large rhodamine cations into the channel is lower than that of small Na^+^ cations. This is probably due to the much larger size of the rhodamine B cations, which can hinder their diffusion and mobility into the channel. In contrast, for the Ag-Gate OECT, results show that the drain current depends slightly on rhodamine concentration (Figure 9b). In particular, the drain current slightly decreases as the concentration increases, which could confirm that rhodamine cations have effectively migrated into the PEDOT:PSS channel. Concerning the gate current, it was observed that it remained constant for the all-PEDOT:PSS OECT (Figure 9c), whereas it slightly increased for the Ag-Gate OECT as a function of both concentration and V_GS_ (Figure 9d), due to the redox reaction (Equation (3)) taking place at the gate electrode with chloride counter-ions present in rhodamine solutions.

In the all-PEDOT:PSS OECTs, cations seem to initially migrate into the channel leading to a slight decrease in the drain current. However, due to the supercapacitor behavior that can be attributable to PEDOT:PSS, the accumulation of charges would simultaneously occur at the surface of both the channel and the gate, which would hinder further migration of cations into the channel. As a result, the channel current remains almost constant and the gate current is kept at very low values. It can therefore be concluded that, for cation detection, the response is maximized in OECTs with non-polarizable gate electrodes (such as Ag). Furthermore, these OECTs exhibit a good response to cations of different sizes, such as Na^+^ and rhodamine.

## 4. Conclusions

OECTs based on PEDOT:PSS were prepared by means of a novel screen-printing method. The electrical characteristics of three types of these screen-printed transistors with different geometries were then compared. OECTs with small ratios between the channel and the gate area showed better current modulation, which is in good agreement with previously published results.

The effect of the gate electrode on the transistor performance for ion-sensing was subsequently investigated by comparing these all-PEDOT:PSS OECTs with non-polarizable Ag-Gate OECTs with the same geometry. The response of these transistors has been studied in aqueous solutions of cations of different sizes (Na^+^ and rhodamine B) with concentrations ranging from 10^−3^ M to 10^−5^ M. The operation of the all-PEDOT:PSS transistors yields a response that is not dependent on ionic concentration. Results suggest that, in these OECTs, few Na^+^ cations have migrated into the channel and that ion migration is further hindered for rhodamine B large-size cations. The weak current modulation of these transistors can be explained assuming that PEDOT:PSS behaves like a supercapacitor. In contrast, Ag-Gate OECTs exhibit a good response to both small Na^+^ cations and large rhodamine B cations. The redox reaction taking place at the gate electrode due to the presence of Cl^−^ counter-ions could explain the strong modulation observed in these devices. It can therefore be concluded that, for cation detection, the use of OECTs with non-polarizable gate electrodes (such as Ag) helps to increase the response.

Results thus confirm that screen-printing can be regarded as a promising alternative technique for the fabrication of low-cost OECTs for cation detection.

## Figures and Tables

**Table 1:** Main characteristics of the CLEVIOS screen-printing inks (Heraeus).

Grade	Application	Resistivity	Viscosity
CLEVIOS S HT	High transparency	1000 Ω/sq	3–5 dPas
CLEVIOS S V3	Standard	700 Ω/sq	15–60 dPas
CLEVIOS S V3 HV	Fine line	700 Ω/sq	60–180 dPas
CLEVIOS S V4	High conductivity	400 Ω/sq	15–60 dPas
